# High-Sensitivity Mass Spectrometry for Probing Gene Translation in Single Embryonic Cells in the Early Frog (*Xenopus)* Embryo

**DOI:** 10.3389/fcell.2016.00100

**Published:** 2016-10-05

**Authors:** Camille Lombard-Banek, Sally A. Moody, Peter Nemes

**Affiliations:** ^1^Department of Chemistry, The George Washington UniversityWashington, DC, USA; ^2^Department of Anatomy and Regenerative Biology, The George Washington UniversityWashington, DC, USA

**Keywords:** single-cell analysis, mass spectrometry, proteomics, cell differentiation, *Xenopus laevis*

## Abstract

Direct measurement of protein expression with single-cell resolution promises to deepen the understanding of the basic molecular processes during normal and impaired development. High-resolution mass spectrometry provides detailed coverage of the proteomic composition of large numbers of cells. Here we discuss recent mass spectrometry developments based on single-cell capillary electrophoresis that extend discovery proteomics to sufficient sensitivity to enable the measurement of proteins in single cells. The single-cell mass spectrometry system is used to detect a large number of proteins in single embryonic cells in the 16-cell embryo of the South African clawed frog (*Xenopus laevis)* that give rise to distinct tissue types. Single-cell measurements of protein expression provide complementary information on gene transcription during early development of the vertebrate embryo, raising a potential to understand how differential gene expression coordinates normal cell heterogeneity during development.

## Introduction

Single-cell analysis technologies are essential to understanding cell heterogeneity during normal development and disease. Characterization of the genomes and their expression at the levels of the transcriptome, proteome, and metabolome provides a molecular window into basic cell processes. Singe-cell measurements complement traditional cell population-averaging approaches by enabling studies at the level of the building blocks of life, where many critical processes unfold (Raj and van Oudenaarden, 2008; Altschuler and Wu, 2010; Singh et al., 2010; Zenobi, 2013). For example, by studying individual cells, it is possible to ask how cells give rise to all the different types of tissues in the body (stem cells) and specialize for defense (immune cells), communication (neurons), and support (glia). This information in turn lays the foundation to developing diagnosis and treatments for addressing pressing health concerns, such as emergence of drug resistant bacteria, onset and development of neurodegeneration, and cancer, as well as infections.

Single-cell investigations take advantage of rapid developments in technology. With more than million-fold amplification of DNA and RNA and the commercialization of high throughput DNA and RNA sequencing, it is now possible to query cell-to-cell differences (Kolisko et al., 2014; Mitra et al., 2014), including but not limited to chromosomal mosaicism in tissues (Vijg, 2014; Gajecka, 2016) and embryonic somatic cells (Liang et al., 2008; Jacobs et al., 2014), establishment of cell heterogeneity in the nervous system (McConnell et al., 2013), and mutations during disease states (Junker and van Oudenaarden, 2015; Kanter and Kalisky, 2015). How gene expression translates into the functionally important proteins and how they then feedback to modulate gene expression is essential to systems cell biology. Multiple reports found differences between transcription and translation (Vogel and Marcotte, 2012; Smits et al., 2014; Peshkin et al., 2015), and transcription is known to be controlled by translational factors during development (Radford et al., 2008); therefore, characterization of the proteome is critical to understanding cell heterogeneity. Translational cell heterogeneity has traditionally been measured by immunohistochemistry and Western blot analyses. Protein-targeted assays have recently gained substantial throughput by the development of mass cytometry (CyTOF), which uses inductively coupled plasma and mass spectrometry (MS) to simultaneously quantify ~35 different proteins tagged with rare earth elements in thousands of cells. This level of multidimensionality has promoted applications in cell differentiation during erythropoiesis (Bendall et al., 2011), and was recently coupled to laser-ablation to spatially survey cell heterogeneity in the tumor environment (Giesen et al., 2014).

Cell heterogeneity has functional implications during embryonic development. Over four decades of innovative embryological manipulations combined with gene-by-gene identifications and functional characterizations in *Xenopus* have shown that molecular asymmetries in the distribution of maternal mRNAs occur upon fertilization and lead to the formation of the three primary germ layers and the germ line (King et al., 2005; Lindeman and Pelegri, 2010). Recent approaches have defined the spatial and temporal changes of mRNAs and abundant proteins and metabolites in the whole embryo (Flachsova et al., 2013; Wuhr et al., 2014; De Domenico et al., 2015). However, very little is known about how these molecules change over time in individual blastomere lineages as they acquire germ layer and body axis fates. In many animals, mRNAs that are synthesized during oogenesis are sequestered to different cytoplasmic domains (Davidson, 1990; Sullivan et al., 2001), which after fertilization then specify the germ cell lineage (King et al., 2005; Haston and Reijo-Pera, 2007; Cuykendall and Houston, 2010) and determine the anterior-posterior and dorsal-ventral axes of the embryo (Heasman, 2006b; Kenyon, 2007; Ratnaparkhi and Courey, 2007; White and Heasman, 2008; Abrams and Mullins, 2009). For example, in *Xenopus* several mRNAs are localized to the animal pole region, which later gives rise to the embryonic ectoderm and the nervous system (Grant et al., 2014), whereas localization of VegT mRNA to the vegetal pole specifies endoderm formation (Xanthos et al., 2001), and region-specific relocalization of the Wnt and Dsh maternal proteins govern the dorsal-ventral patterning of the embryo (Heasman, 2006a; White and Heasman, 2008). However, there is abundant evidence that in developing systems not all transcripts are translated into proteins; therefore, analyses of the mRNAs may not reveal the activity state of the cell. In fact, different animal blastomeres of the 16-cell *Xenopus* embryo that are transcriptionally silent can have very different potentials to give rise to neural tissues (Gallagher et al., 1991; Hainski and Moody, 1992; Yan and Moody, 2007), even though they appear to express common mRNAs (Grant et al., 2014; Gaur et al., 2016).

High-resolution MS is the technology of choice for the analysis of the proteome (Aebersold and Mann, 2003; Guerrera and Kleiner, 2005; Walther and Mann, 2010; Zhang et al., 2013). Using millions of cells, contemporary MS enables the discovery (untargeted) characterization of the encoded proteomes of various species in near complete coverage, as recently demonstrated for the yeast (Hebert et al., 2014), mouse (Geiger et al., 2013), and human (Wilhelm et al., 2014). Recent whole-embryo analyses by MS revealed that transcriptomic events are accompanied by gross proteomic and metabolic changes during the development of *Xenopus* (Sindelka et al., 2010; Vastag et al., 2011; Flachsova et al., 2013; Shrestha et al., 2014; Sun et al., 2014), raising the question whether these chemical changes are heterogeneous also between individual cells of the embryo at different embryonic developmental stages. However, the challenge has been to collect high-quality signal from the miniscule amounts of molecules contained within single blastomeres for analysis. Since different blastomeres in *Xenopus* are fated to give rise to different tissues (Moody, 1987a,b; Moody and Kline, 1990), elucidating the proteome in individual cells of the embryo holds a great potential to elevate our understanding of the cellular physiology that regulates embryogenesis. For a deeper understanding of the developmental processes that govern early embryonic processes, it would be transformative to assay the ultimate indicator of gene expression downstream of transcription: the proteome.

To address this cell biology question, we and others have developed platforms to extend MS to single cells (see reviews in References Mellors et al., 2010; Rubakhin et al., 2011; Passarelli and Ewing, 2013; Li et al., 2015). For example, targeted proteins have been measured in erythrocytes (Hofstadler et al., 1995; Valaskovic et al., 1996; Mellors et al., 2010). Discovery MS has been used in the study of protein partitioning in the nucleus of the *Xenopus laevis* oocyte (Wuhr et al., 2015). Recently, we have developed single-cell analysis workflows and custom-built microanalytical capillary electrophoresis (CE) platforms for MS to enable the discovery (untargeted) characterization of gene translation in single embryonic cells (blastomeres). Using single-cell CE, we have measured hundreds–thousands of proteins in blastomeres giving rise to distinct tissues in the frog (*X. laevis*), such as neural, epidermal, and gut tissues (Moody, 1987a). We have also established quantitative approaches to compare gene translation between these cell types. Quantification of ~150 different proteins between the blastomeres has captured translational cell heterogeneity in the 16-cell vertebrate embryo (Lombard-Banek et al., 2016a). These results complement known transcriptional cell differences in the embryo, but also provide previously unknown details on how differential gene expression establishes cell heterogeneity during early embryonic development.

**Figure 1:**
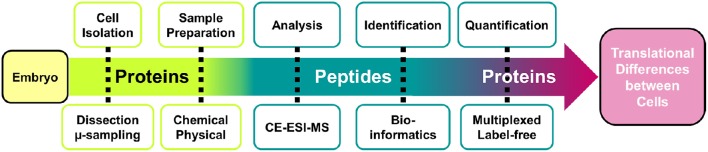
**Analytical workflow for the bottom-up measurements of protein expression in single embryonic cells**. A custom-built high-sensitivity capillary electrophoresis electrospray ionization mass spectrometer (CE-ESI-MS) is used to identify and quantify proteins.

In this contribution, we give an overview of the major steps of the single-cell CE-MS workflow (Figure 1). Protocols are provided to isolate single cells, extract and process proteins, and use the CE-MS platform to identify and quantify protein expression. Additional details on technology development and validation are available elsewhere (Nemes et al., 2013; Onjiko et al., 2015; Lombard-Banek et al., 2016a,b). These protocols have allowed us to study proteins (Lombard-Banek et al., 2016a,b) and metabolites (Onjiko et al., 2015, 2016) in single blastomeres in 8-, 16-, and 32-cell *X. laevis* embryos. Additionally, trouble-shooting advice (Table 1) is provided to help others adopt single-cell MS toward the systems biology characterization of molecular processes in cells and limited amounts of specimens.

**Table 1:** **Troubleshooting advice for CE-ESI-MS for bottom-up proteomics**.

**Issues**	**Potential causes**	**Advice**
No peptides detected	Failed enzymatic digestion	Repeat analysis; if problem persists, repeat protein digestion (use standard proteins as quality control)
CE current drops drastically	Capillary is clogged or a bubble was injected	Flush the capillary with the BGE for ~10–15 min; repeat analysis
Electrospray is unstable	Electrolysis in the CE-ESI interface; the sheath flow connection is loose	Lower the spray voltage; revise connections; repeat analysis
Low number of protein identifications	Erroneous injection; inaccurate calibration of the mass spectrometer	Repeat analysis; calibrate the mass spectrometer

## Materials and equipment

### Single blastomere dissection

- Fine sharp forceps (e.g., Dumont #5). One forceps should have a squared tip, while the other should be sharpened to a fine tip.
- Sterile Pasteur pipets.
- Hair loop: place a fine hair (~10 cm long) into a 6″ Pasteur pipet to form a 2–3 mm loop and secure it in place with melted paraffin. Sterilize the hair loop before usage by dipping it in 70% methanol.
- 0.6 mL centrifuge tubes.
- 60 and 90 mm Petri dishes.
- Incubator set to 14°C.
- Dejellying solution: 2% cysteine hydrochloride in water, pH 8, prepared by adding 20 g of crystalline cysteine hydrochloride into 1 L of distilled water. pH is adjusted to 8 by adding 10 N NaOH drop-wise.
- 100% Steinberg's solution (SS): Dissolve the following salts into 1 L of distilled water: 3.5064 g NaCl, 49.9 mg KCl, 99.9 mg MgSO_4_, 55.8 mg Ca(NO_3_)_2_, 0.6302 g Tris-HCl, and 80.0 mg Tris-base. Adjust the pH to 7.4. Autoclave and store in 14°C incubator.
- 50% Steinberg's solution: Dilute 50 mL of 100% SS with 50 mL of distilled water.
- Dissection dish: add 2 g of agarose in 100 mL of 100% Steinberg's solution. Dissolve the agarose by autoclaving. Once the bottle is cool enough to handle, pour the agarose mixture to ~1 mm in thickness into 60 mm in diameter Petri dishes. Alternatively, the agarose mixture can be stored at 4°C, and reheated in a microwave before use. Dishes should be stored wrapped in plastic at 4°C to prevent dehydration of the agarose.
- *X. laevis* (adult male and female). Protocols related to the handling and manipulation of animals must adhere to Institutional and/or Federal guidelines; the work reported here was approved by the George Washington University Institutional Animal Care and Use Committee (IACUC #A311).

### Protein extraction, enzymatic digestion, and quantification

- Refrigerated centrifuge (4°C)
- Heat blocks (2) set to 60 and 37°C.
- A −20°C freezer.
- Sonication bath (e.g., Brandson CPX 2800).
- A vacuum concentrator (e.g., CentriVap, LabConco).
- Lysis buffer: for 1 mL of lysis buffer, mix 100 μL of 10% sodium dodecyl sulfate (SDS), 100 μL of 1.5 M NaCl, 20 μL of 1 M Tris-HCl (pH 7.5), 10 μL of 0.5 M EDTA, and 770 μL of H_2_O.
- Enzymatic digestion solution, 50 mM ammonium bicarbonate: add 0.1976 g of crystalline ammonium bicarbonate to HPLC grade water.
- Dithiothreitol (1 M): Dissolve 0.1543 g of solid dithiothreitol into 1 mL of 50 mM ammonium bicarbonate. Divide in 50–100 μL aliquots and store at −20°C for months.
- Iodoacetamide (1 M): Dissolve 0.1850 g of crystalline iodoacetamide into 1 mL of 50 mM ammonium bicarbonate. Iodoacetamide is light sensitive and therefore should be kept away from any light sources. It is suggested to make freshly before use, but storage in 50–100 μL aliquots at −20°C is acceptable for up to 2 months. Aliquots are only for single use, do not freeze-thaw.
- Trypsin solution 0.5 μg/μL: dissolve a 20 μg vial in 40 μL of 1 mM HCl in water.
- Tandem mass tags kit (e.g., TMT10plex, Thermo Scientific).

### CE-ESI-MS analysis

- HPLC grade solvents and reagents: water, acetonitrile, methanol, formic acid, and acetic acid.
- Regulated high voltage power supplies (2) outputting up to 5 kV for maintaining the electrospray (e.g., P350, Stanford Research Systems), and up to 30 kV for CE separation (e.g., Bertan 230-30R, Spellman).
- Separation capillary: 40/110 μm (i.d./o.d.) bare fused silica capillary from Polymicro.
- Sample solvent: mix 500 μL methanol with 500 μL water and 0.5 μL acetic acid.
- Sheath solution: add 50 mL of methanol to 50 mL of water and 50 μL of formic acid.
- Background electrolyte: to prepare 50 mL, mix 12.5 mL of acetonitrile, and 1.887 mL of formic acid with 35.613 mL of water.
- High-resolution mass spectrometer (e.g., Orbitrap Fusion, Thermo).

## Procedures

### Sample preparation

**Figure 2:**
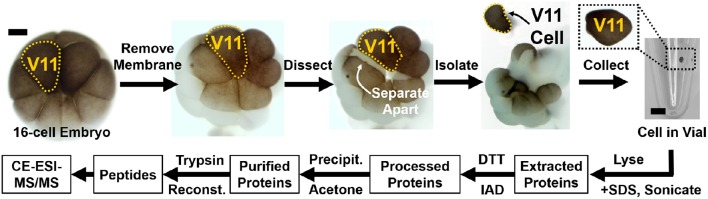
**Isolation of identified cells and processing of their protein content**. Example shows how the epidermal-fated ventral-animal cell (named V11) was identified in the 16-cell *X. laevis* embryo based on pigmentation, cell size, and location in reference to established cell fate maps (Moody, 1987a). The cell was processed via bottom-up proteomic workflow, and the resulting peptides collected for proteomic analysis. Key: DTT, dithiothreitol; IAD, iodoacetamide. Scale bar = 200 μm (embryo), 1.25 mm (vial).

The goal of sample preparation is to extract proteins from single cells and process the proteins for MS analysis. The workflow (Figure 1) starts with the identification of blastomeres in the embryo in reference to established cell fate maps (Moody, 1987a,b; Moody and Kline, 1990; Lee et al., 2012) and differences in cell size and pigmentation. Cells are microdissected using sharp forceps and collected into individual microcentrifuge tubes. Figure 2 shows the dissection of the V11 cell. Next, isolated blastomeres are lysed using chemical (detergent) and physical (ultrasonication) methods, and their proteins are extracted. The proteins are processed via standard bottom-up proteomics protocols (Zhang et al., 2013), whereby reduction, alkylation, and enzymatic digestion are performed to convert proteins into peptides that are more readily analyzable by MS.

#### Single blastomere dissection and isolation

As detailed protocols are available on the identification and dissection of blastomeres (Moody, 2012; Grant et al., 2013), only a brief summary of the major steps follows.

1. Prepare consumables:
  - 2% cysteine solution
  - 100% Steinberg solution (SS)
  - 50% Steinberg solution (SS)
  - Sterile Pasteur pipet
  - Petri dish filled with 2% agarose (w/v in 100% SS)
  - Sharp forceps
  - Hair loop
  - 0.6 mL microcentrifuge tubes
2. Remove jelly coats that naturally surround the embryos:
  - Add 4 × volume of the cysteine solution to the embryos (Table 2) and gently swirl the solution for ~4 min.
  - Once the embryos are free of the jelly coat, immediately wash them with 100% SS (Table 2) 4 times for 2 min each.
  - Transfer the embryos to a clean Petri dish filled with 100% SS and store them at 14–20°C in an incubator.
3. Dissect cells from the embryos as published elsewhere (Grant et al., 2013). A representative example is shown in Figure 2. Briefly:
  - Transfer the selected embryos to a 60 mm Petri dish coated with 2% agarose and filled with 50% SS.
  - Place the embryo of interest in a groove made in the agarose coating.
  - Orient the embryo for easy handling of the cell of interest using a hair loop.
  - Remove the vitelline membrane gently using sharp forceps. During this step, take care not to damage the embryo.
  - Hold the embryo using sharp forceps on the opposite side of the cell of interest, and gently pull on either side to isolate the cell.
  - Transfer isolated cells using a sterile Pasteur pipet into a micro-centrifuge tube.

### Protein extraction and enzymatic digestion

1. Prepare consumables:
  - Lysis buffer
  - Acetone chilled to −20°C
  - 50 mM ammonium bicarbonate
  - 1 M dithiothreitol
  - 1 M iodoacetamide
  - Sonication bath (e.g., Brandson CPX 2800)
2. Lyse the cells to release their content:
  - Remove the excess 50% SS from around the cell. Take care not to disrupt the cell.
  - Add 10 μL of lysis buffer (Table 2) and vortex for ~30 s.
  - Sonicate for ~5 min, vortex for ~30 s. Repeat this step 3 times.
  - (Optionally) Add protease inhibitor to the lysis buffer to minimize/avoid protein degradation during this step.
3. Reduce and alkylate protein disulfide bonds:
  - Add 0.5 μL of 1 M dithiothreitol to the sample, and incubate for 20–30 min at 60°C.
  - Add 1 μL of 1 M iodoacetamide and incubate for 15 min in the dark at room temperature.
  - Quench the reaction by adding 0.5 μL of 1 M dithiothreitol.
4. Purify proteins by cold acetone precipitation.
  - Add to the cell extract a volume of pure acetone that is 5 times that of the cell extract (~50 μL), and incubate at −20°C overnight.
  - Recover the precipitated proteins by centrifugation at 10,000 × g for 10 min and 4°C.
  - Remove the supernatant.
  - Dry the pellet using a vacuum concentrator.
  - (Optional) Store the protein pellet at –20 or −80°C for up to 3 months.
5. Digest proteins for bottom-up proteomics analysis. A variety of enzymes or a combination of enzymes can be used for this task (e.g., trypsin, lysine C). We choose trypsin due to its benefits for MS analysis (Zhang et al., 2013).
  - Reconstitute the protein pellet in 50 mM ammonium bicarbonate.
  - Add 0.3 μL of 0.5 μg/μL trypsin (trypsin in 1 mM HCl), equivalent to a protease/protein ratio of ~1/50.
  - Incubate overnight at 37°C.
6. (Optional) Store the digest at −80°C for up to 3 months.

**Table 2:** **Solutions and their uses**.

**Solution/buffer**	**Composition**	**Usage**	**Storage conditions**
Cysteine Hydrochloride	2% (w/v) cysteine hydrochloride, pH 8 adjusted with 10 N NaOH drop wise	Removes the jelly coats surrounding embryos	Make fresh
Steinberg's Solution (SS)	60 mM NaCl, 0.67 mM KCl, 0.83 mM MgSO_4_, 0.34 mM Ca(NO_3_)_2_, 4 mM Tris–HCl, 0.66 mM Tris base, in distilled water, pH 7.4. Autoclaved. Store in incubator for months.	Provides media for culturing embryos	4–14°C
Lysis Buffer	1% sodium dodecyl sulfate (SDS), 150 mM NaCl, 20 mM Tris-HCl pH 8, 5 mM EDTA in distilled water	Lyses cells/tissues	4°C
Sample Solvent	50–60% acetonitrile in water, 0.05% acetic acid (all solvents are LC-MS grade)	Reconstitutes protein digest	4°C
Background Electrolyte (BGE)	25% acetonitrile in water, 1 M formic acid (all solvents are LC-MS grade)	Electrolyte for CE	4°C
Electrsopray Sheath Liquid	50% methanol in water, 0.1% formic acid (all solvents are LC-MS grade)	Stabilizes ESI-MS operation	4°C

#### Quantification

The presented technology is compatible with well-established protocols in quantitative proteomics. Stable isotope labeling with amino acids in cell culture (SILAC) allows barcoding of proteins with isotopic labels for multiplexing quantification (Geiger et al., 2013). Label-free quantification (LFQ) is an alternative strategy whereby peptide signal abundance is used as a proxy for protein concentration. We have recently demonstrated LFQ for single blastomeres of neural fates in the 16-cell embryo using the protocol presented here (Lombard-Banek et al., 2016b). Alternatively, relative quantification can be performed using designer mass tags. In this approach, proteins are digested to peptides and the peptides barcoded with isotopic labels that can be distinguished by high-resolution MS. Multiple protocols allow for quantifying protein expression at the level of peptides in high throughput via multiplexing, including tandem mass tags (TMT) (Thompson et al., 2006; McAlister et al., 2014), and isobaric tag for relative and absolute quantitation (iTRAQ; Ross et al., 2004), and di-Leu (Xiang et al., 2010; Frost and Li, 2016). We have recently downscaled TMT-based multiplexed quantification to the protein content of single blastomeres using the following strategy (adapted from the vendor), which we then used to compare protein expression between the D11, V11, and V21 cells (Lombard-Banek et al., 2016a) that are fated to give rise to different types of tissues (neural, epidermal, and hindgut, respectively):

- Add 15 μL of TMT reagent to each digest and incubate for 1 h at room temperature.
- Add 3.5 μL of hydroxylamine and incubate for 15 min at room temperature.
- Mix the samples together at a 1:1 ratio (volume or total protein content)
- Dry the sample using a vacuum concentrator.
- Add 5 μL of 60% acetonitrile containing 0.05% formic acid.

### Sample analysis using CE-ESI-MS

**Figure 3:**
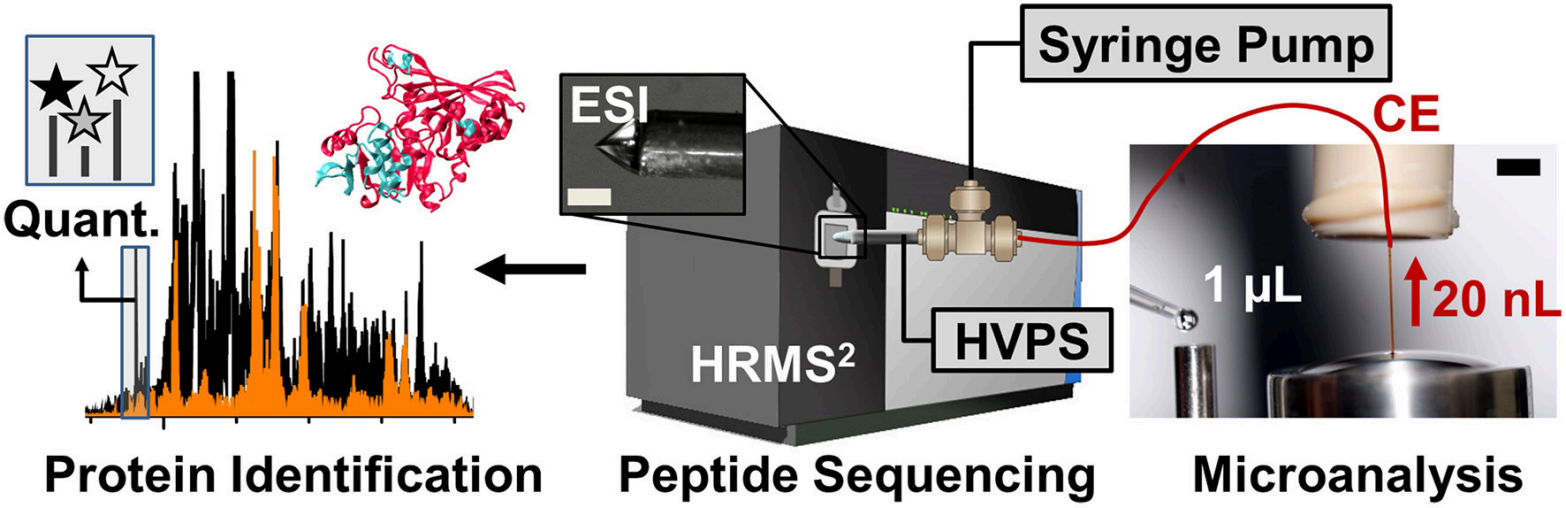
**Schematics of the high-sensitivity proteomic analyzer**. The platform integrates microanalytical capillary electrophoresis (CE), electrospray ionization (ESI), and high-resolution tandem mass spectrometry (HRMS^2^). Scale bar = 150 μm (ESI), 1.5 mm (CE panel). Key: HVPS, high-voltage power supply. Figure adapted with permission from Lombard-Banek et al. (2016a).

**Figure 4:**
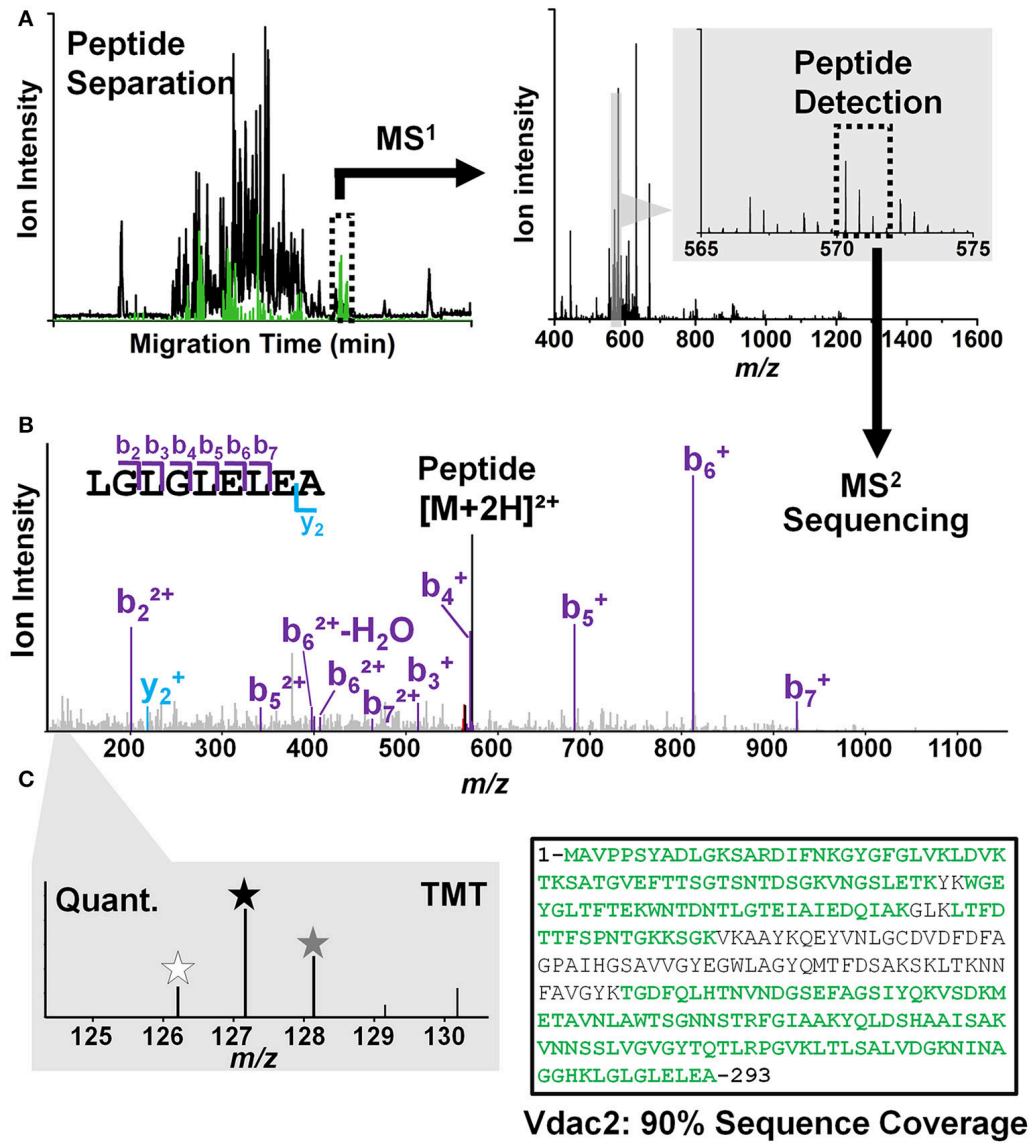
**Peptide identification/quantification in CE-ESI-HRMS^2^ using a bottom-up strategy. (A)** Peptides are electrophoretically separated (left panel) and their accurate mass is measured (right panel). **(B)** Peptide signals are sequenced by tandem MS (MS^2^). For example, a signal was detected with *m/z* 572.33 at ~50 min separation, which was assigned to the sequence LGLGLELEA based on the MS^2^ data. **(C)** Peptides are quantified and assigned to the source protein. Tandem mass tags (TMT) with different *m/z* values (indicated by asterisks of different color in left panel) are used to barcode peptides from different cells, allowing their simultaneous analysis (multiplexing) with higher throughput (left panel). For example, the sequence LGLGLELEA was unique to the voltage-dependent anion channel 2 protein in the *Xenopus* proteome. The presence of other peptides allowed identifying this protein in high sequence coverage; see detected sequence in green (right panel).

Peptides are analyzed using a custom-built CE-ESI-MS platform (Nemes et al., 2013; Onjiko et al., 2015; Lombard-Banek et al., 2016a). Instructions regarding the construction and operation of the platform are available from elsewhere (Nemes et al., 2013). Schematics of the CE-ESI-MS instrument are shown in Figure 3. CE is selected to electrophoretically separate peptides in a fused silica capillary by applying voltage difference across the capillary ends. As a general rule, peptides with smaller size and higher charge state migrate faster through the capillary. A high resolution mass spectrometer is used to sequence peptides via data-dependent acquisition. In this approach, eluting peptides are detected based on single-stage (full) scans (MS^1^) and are sequenced by tandem-MS (MS^2^ scans) using collision-induced dissociation (CID), higher-energy collisional dissociation (HCD), or other fragmentation technologies. The tandem mass spectra reveal sequence information for the peptides, as also exemplified for LGLGLELEA in Figure 4. During quantification experiments, the TMT labels also dissociate from the peptide, and the relative abundance of these TMT signals serves as quantitative measure of protein abundance (Figure 4C, right panel).

#### CE-ESI-MS measurements

1. Build the CE-ESI-MS system as described elsewhere (Nemes et al., 2013; Onjiko et al., 2015). For bottom-up proteomics of single *Xenopus* blastomeres, operate the system as recently established (Lombard-Banek et al., 2016a,b).
2. Prepare the CE system ~15 min prior to start the experiments as follow:
  - Flush the capillary with background electrolyte (25% acetonitrile with 1 M formic acid).
  - Flush the sheath capillary with electrospray solution (50% methanol with 0.1% formic acid)
  - Turn on the electronics (high voltage power supplies, syringe pumps, mass spectrometer, etc.) for ~30 min to stabilize operation.
3. Inject the sample into the capillary as follows:
  - Transfer the capillary into the background electrolyte vial.
  - Deposit ~1 μL of sample onto the sample microvial (see Figure 3).
  - Transfer the capillary from the BGE vial to the sample vial.
  - Elevate the injection stage by ~15 cm for ~3 min to siphon ~20 nL of the sample into the CE capillary.
  - Lower the injection stage to level the capillary inlet to the outlet, and transfer the capillary inlet end into the BGE vial.
  - Apply ~10,000 V to the background electrolyte vial to start electrophoretic separation of the peptides.
  - Increase the electrospray voltage gradually until the cone jet mode is established for efficient ionization (Nemes et al., 2007). Using a long-distance microscope, carefully inspect the electrospray emitter to avoid electrical breakdown; electrical discharge, spark, or arc risks the mass spectrometer. In our experiments, the electrospray emitter is positioned ~0.5 cm from the mass spectrometer orifice and is biased to 3000 V to generate the cone-jet spray.
  - Ramp the separation voltage to ~18,000 V. In our system, we limit the separation voltage to keep the CE current < 8 μA to prevent/minimize electrolysis or solvent heating. Monitor the CE current and adjust the separation voltage as necessary. For instructions on how to measure the current, refer to Nemes et al. (2013).
  - Start MS acquisition with data-dependent acquisition as specified by the mass spectrometer vendor. For example, we use the following settings for a quadrupole-orbitrap linear ion trap mass spectrometer (Fusion, Thermo Scientific): MS^1^ analyzer resolution (orbitrap), 60,000 FWHM; *m/z* scan range, 350–1600; injection time, 100 ms; precursor ion selection window, 0.8 Da in the quadrupole cell; fragmentation, HCD with 30% normalized energy in the multipole cell using nitrogen collision gas; MS^2^ analyzer rate, rapid scan; MS^2^ maximum injection time, 50 ms.

#### Protein identification

Last, peptide sequences are compared to the proteome of the specimen (*X. laevis* here) to identify proteins (see Figure 4). This step is facilitated by readily available proteomes from SwissProt, UniProt, and experimentally determined RNA expression (Wang et al., 2012; Smits et al., 2014; Wuhr et al., 2014). Well-established bioinformatics software packages are used to process raw mass spectrometric data. For example, Proteome Discoverer (Thermo Scientific), ProteinScape (Bruker Daltonics), and MaxQuant (Cox and Mann, 2008) interpret MS–tandem-MS datasets by executing well-established search engines, such as SEQUEST (Eng et al., 1994), Mascot (Perkins et al., 1999), and Andromeda (Cox et al., 2011). The general strategy of bottom-up proteomics has recently been reviewed in detail (Sadygov et al., 2004; Cox et al., 2011; Zhang et al., 2013). We typically acquire tens of thousands to a million mass spectra, which identify 2000–4000 peptides in single blastomeres in the 16-cell embryo. These data allow us to identify ~1700 protein groups and quantify hundreds of proteins between the D11, V11, and V21 cells.

### Anticipated results

**Figure 5:**
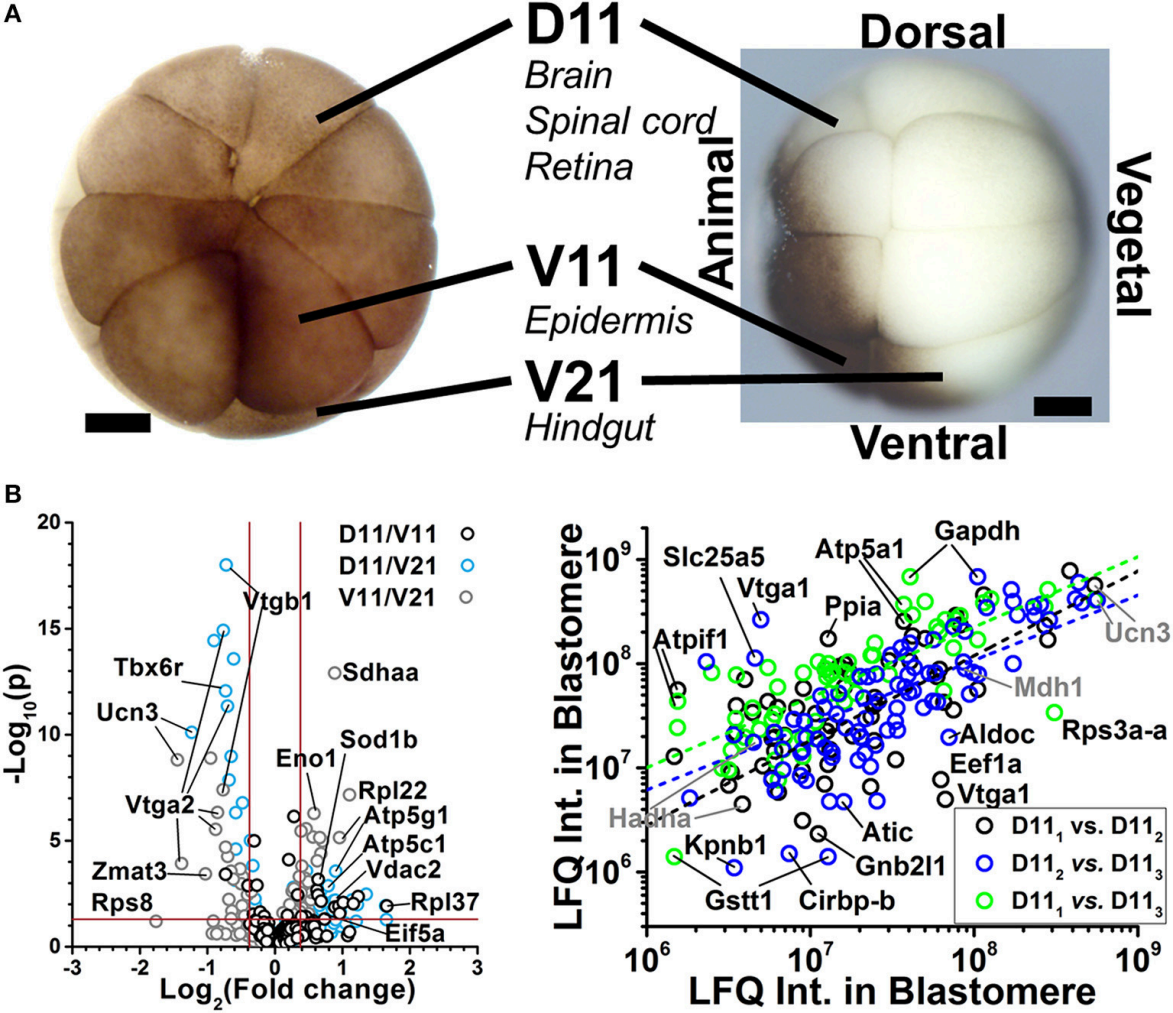
**Examples of protein identification–quantification between single embryonic cells. (A)** The D11, V11, and V21 cells have different tissues fates in the frog *X. laevis*. Scale bars: 250 μm. Figure reprinted with permission from Onjiko et al. (2015). **(B)** These cells were dissected from different 16-cell *X. laevis* embryos and analyzed using multiplexed (left panel) and label-free quantification (right panel). Volcano plots reveal gene translation differences between the V11, D11, and V21 cell types (left). Pearson correlation analysis of protein expression finds similar protein expression for the majority of proteins between D11 blastomeres, and detectable differences for others (right panel). Figures adapted with permission from Lombard-Banek et al. (2016a,b).

The CE-ESI-MS can be used to identify gene translational differences between cells. As shown in Figure 5, we have used this approach to assess protein differences between blastomeres of the 16-cell *X. laevis* embryo (Lombard-Banek et al., 2016a,b). Cell types with different tissue developmental fates were analyzed: the midline dorsal-animal cell (named D11) develops mainly into the retina and brain, the midline ventral-animal cell (named V11) gives rise primarily to the head and trunk epidermis, and the midline ventral-vegetal cell (named V21) is the primary precursor of the hindgut. The approach allowed the identification of 1709 protein groups (< 1% false discovery rate, FDR) from ~20 ng of protein digest, corresponding to ~0.2% of the total protein content of the blastomere (Lombard-Banek et al., 2016a). Many of the identified proteins are known to be involved in different cell fates. For example, Geminin (Gem) and Isthmin (Ism) were detected in the D11 cells in our measurements, and these proteins are involved in brain development (Pera et al., 2002; Seo et al., 2005), which is the stereotypical fate of D11 cells (Moody, 1987a). Multiplexed quantification by TMTs provided comparative evaluation for 152 non-redundant protein groups between the cell types (Figure 5B, left), including many that were significantly differentially expressed between the cell types (*p* < 0.05, fold change ≥1.3). We have also performed label free quantitation (LFQ) to compare D11 cells that were isolated at similar developmental phase of the 16-cell *X. laevis* embryos (Figure 5A). A Pearson correlation analysis showed similar expression levels for the majority of proteins between the D11 cells (see proteins along linear fits). The study also found 25 proteins that were differentially accumulated in the respective cells, suggesting highly variable expression (Figure 5B, right; Lombard-Banek et al., 2016b). These data on translational cell heterogeneity complement transcriptomic information on cell differences (Flachsova et al., 2013), but also provide new insights into how differential gene expression sets up different cell fates and the major developmental axes of the early embryo.

## Conclusions

High-sensitivity MS enables the identification and quantification of a sufficiently large number of proteins to study cell and developmental processes at the level of individual cells. Advances in sampling (smaller single cells), protein processing, microanalytical MS, and bioinformatics have enabled the discovery characterization of hundreds to thousands of proteins in single cells. Unbiased measurement of protein translation by MS complements genomic and transcriptomic information, essentially laying down the foundation of the molecular characterization of cell heterogeneity. Knowledge of genomic, transcriptomic, proteomic, and metabolomic processes paves the way to understanding how differential gene expression establishes cell heterogeneity during normal development and disease states.

## Author contributions

CL, SM, and PN wrote the manuscript.

## Funding

This research was supported by National Science Foundation Grant DBI-1455474 (to PN and SM) and the George Washington University Start-Up Funds (to PN) and Columbian College Facilitating Funds (to PN and SM). The content of the presented work was solely the responsibility of the authors and does not necessarily represent the official views of the funding agencies.

### Conflict of interest statement

The authors declare that the research was conducted in the absence of any commercial or financial relationships that could be construed as a potential conflict of interest.
